# Gallbladder agenesis: a systematic review of presentation, management, and outcomes

**DOI:** 10.1097/MS9.0000000000005084

**Published:** 2026-04-29

**Authors:** Erin N. Young, Ghyslaine Bruna Djeunang Dongho, Ngo Valery Ngo, Hilary M. Jasmin, Solange Dabou, Regina Sinsai, Constantine T. Asahngwa, Ronald M. Gobina, Denis A. Foretia

**Affiliations:** a College of Medicine, University of Tennessee Health Science Center, Memphis, TN, USA; b Division of Health Policy and Research, Nkafu Policy Institute, Yaoundé, Cameroon; c Center for Evidence-based Policy, Oregon Health and Science University, Portland, OR, USA; d Department of Anthropology, University of Yaoundé 1, Yaoundé, Cameroon; e Buea Regional Hospital, South West Region, Cameroon; f Department of Surgery, Virginia Tech Carilion School of Medicine, Roanoke, VA, USA

**Keywords:** biliary disease, gallbladder agenesis, surgical complications, systematic review

## Abstract

**Introduction:**

Gallbladder agenesis (GA) is often misdiagnosed as a gallbladder pathology. This systematic review aimed to summarize the current evidence on epidemiology, clinical presentation, diagnostic approaches, and management strategies.

**Methods:**

PubMed/MEDLINE, Scopus, and Embase databases were comprehensively searched in English, French, and Spanish for GA cases published before November 2022. Two reviewers screened articles using the Preferred Reporting Items for Systematic Reviews and Meta-Analyses guidelines. Data were analyzed to identify the clinical features, diagnostic approaches, management strategies, and associated factors.

**Results:**

A total of 9186 unique articles were screened, 125 of which met the inclusion criteria (151 cases in total). Most patients were female (66%), with a median age of 45 years. Most patients (73%) were diagnosed intraoperatively, and the most common presenting symptoms were right upper quadrant pain (66%), nausea (32%), or emesis (30%). Less than 15% of patients had elevated liver enzyme levels or leukocytosis. Ultrasonography was the primary imaging modality (81%). Most patients (79%) underwent operative management, with laparoscopy in 49.6% and exploratory laparotomy in 50.4%. Diagnosis by hepatobiliary iminodiacetic acid scan was associated with higher complications (odds ratio 8.53, 95% confidence interval 1.21–59.1, *P* = 0.026). There were more complications in the US cohort than in the non-US cohort (20% vs. 4.5%, *P* = 0.006).

**Conclusions:**

This systematic review showed that many patients with GA underwent unnecessary operative interventions with an associated increase in complications. Surgeons should be familiar with this diagnosis to minimize unnecessary interventions and reduce healthcare costs.

## Introduction

Gallbladder agenesis (GA) is a well-recognized but rare condition in which the gallbladder and cystic duct fail to develop, and are subsequently not found at the usual or most common atypical sites. It was first described in 1701 by Lemery, with over a hundred case reports published since then^[^1^]^. The condition is present in 0.0027–0.007% of the population and is generally accepted as a congenital malformation^[^2,3^]^. The etiology is unknown, but multiple family members have been reported to have the diagnosis^[^4–7^]^. The gallbladder primarily stores bile and facilitates fat digestion^[^8^]^. Gallbladder disease has a varied presentation and includes biliary colic, cholelithiasis, symptomatic cholelithiasis, cholecystitis, and carcinoma^[^9^]^. Patients with agenesis of the gallbladder can present with classic biliary symptoms or are found incidentally on imaging^[^10^]^.

HIGHLIGHTS

- Gallbladder agenesis is the congenital absence of the gallbladder.
- Patients often present with symptoms mimicking biliary pathology.
- It is frequently misdiagnosed preoperatively, leading to unnecessary operative interventions.
- Surgeons must avoid unnecessary exploration of the biliary system in search of a non-existent gallbladder.

While the use of modern imaging has improved the accuracy of diagnosis, many cases are still missed, thus subjecting patients to unnecessary interventions^[^11^]^. Additional imaging such as cholangiography, computed tomography (CT), endoscopic retrograde cholangiopancreatography, or magnetic resonance cholangiography are often necessary when ultrasound findings are inconclusive^[^12^]^. However, there are reports of intraoperative searches for a missing gallbladder with potential injury to the biliary system^[^2,13^]^.

To date, no comprehensive review of GA has captured its epidemiology, clinical presentation, diagnostic approaches, and management strategies. This systematic review aimed to summarize the current knowledge of GA, including its epidemiology, clinical presentation, diagnostic approaches, and management strategies.

## Methods

This systematic review followed the Preferred Reporting Items for Systematic Reviews and Meta-Analyses statement guidelines^[^14^]^. The inclusion criteria were as follows: full-text articles published in English, French, or Spanish up to November 2022 describing cases of GA in both adult and pediatric individuals aged greater than 1 year; diagnosis confirmed either by imaging or intraoperatively. Animal studies were excluded from analysis. A librarian developed a search strategy and browsed PubMed/MEDLINE, Scopus, and Embase for results, including “gallbladder agenesis,” “absent gallbladder,” and “agenesis of the gallbladder.” Table 1 shows the search strategy used for PubMed. This strategy was adopted to meet the syntax-specific requirements of each database.

**Table 1:** MEDLINE/PubMed search strategy.

Databases	Search strategy
MEDLINE/PubMed	(((((Gallbladder, Agenesis Of [Supplementary Concept] OR Congenital Abnormalities [Mesh]) OR ((birth[tw] AND defect[tw]) OR (fetal[tw] AND (anomal*[tw] OR malform*[tw])))) OR (congenital[tw] AND (abnormality[tw] OR abnormalities[tw] OR defect*[tw]))) OR (agenesis[tw])) OR ((congenital[tw] AND abnormality[tw]))) AND ((cystic[tw] AND duct[tw]) OR (gallbladder[tw]) OR Cystic Duct[Mesh] OR “gallbladder”[MeSH Terms])

The authors, 2022.

**Figure 1.**
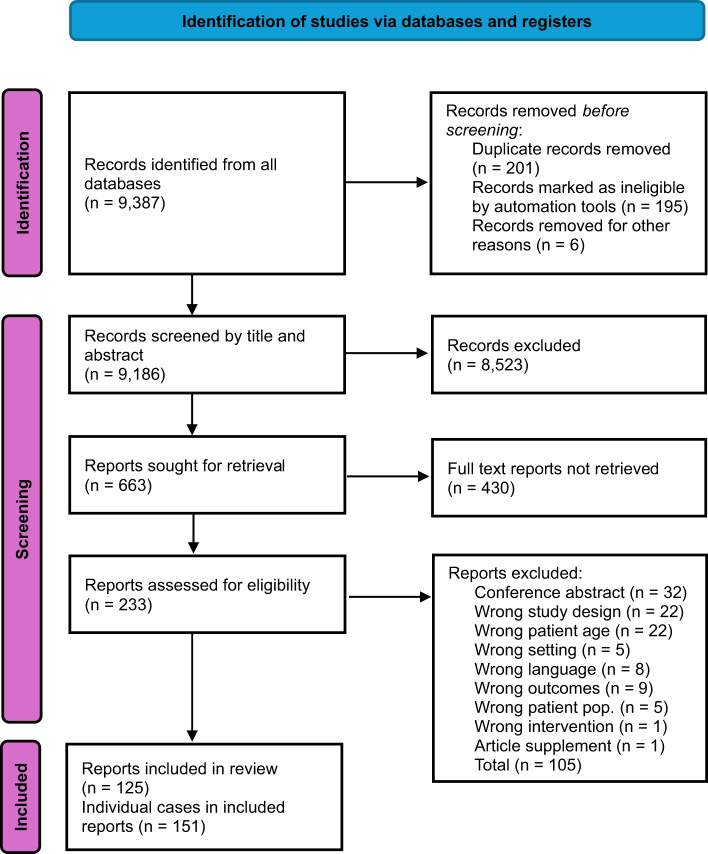
PRISMA flow chart detailing the search strategy.

Appropriate cases were selected for these three phases. We searched all databases, imported the results to EndNote 20 for deduplication, and transferred the records to Covidence, a free online record screening program. Two independent reviewers screened titles and abstracts in the second stage to determine their eligibility. Finally, two reviewers evaluated the full-text articles to confirm that they met the inclusion criteria. Articles excluded during screening and full-text review were documented along with the reasons for exclusion. Discrepancies between the two reviewers were resolved by consensus or by a third independent reviewer. Because the evidence base comprised of case reports and few case series with descriptive designs, methodologically quality focused on including only cases confirmed by imaging or intraoperatively. Two reviewers independently assessed each record using piloted forms, with disagreements resolved by a third reviewer. The domains included (i) ascertainment (case definition) and (ii) information bias (completeness and clarity of clinical history, imaging, interventions, and outcome measurement). There was no consideration for alternative explanations as patients with a surgically absent gallbladder were not eligible as a case. In accordance with the PRISMA 2020 statement, we prepared a flowchart depicting the process followed in this systematic review (Fig. 1).

Two independent reviewers extracted data using a predesigned data extraction form. Any conflicts between the reviewers’ data were resolved by consensus or by a third independent reviewer. The complete dataset included age, sex, associated congenital anomalies, presenting symptoms, laboratory results, diagnostic imaging, operative interventions (laparoscopy, laparotomy, etc.), postoperative complications, and 30-day mortality outcomes. We included only cases with sufficient data and quality based on availability of the above information.

Assessment of heterogeneity: Given that the dataset consisted of individual case reports and small case series with sparse, inconsistently reported variables, we assessed heterogeneity narratively rather than statistically. We compared clinical and methodological features across reports (such as patient characteristics, diagnostic modality, surgical approach, publication year) and presented stratified descriptive summaries where data permitted (such as pre- vs. intraoperative diagnosis or laparoscopic vs. open management). Because the data did not support valid quantitative pooling, we did not estimate Cochran’s *Q*, τ^2^, *I*^2^, prediction intervals, or conduct subgroup meta-analyses or meta-regression.

We summarized categorical variables as counts and percentages and continuous variables as medians (or means with standard deviations, as appropriate). Group comparisons for categorical outcomes used Fisher’s exact test (chi-square applied only when all expected cell counts were ≥5), and continuous outcomes were compared with the Wilcoxon rank-sum test. Where applicable, effect sizes are presented as odds ratios (ORs) with 95% confidence intervals (CIs), and two-sided *P* < 0.05 was considered statistically significant. We fitted a logistic regression model to estimate adjusted associations between prespecified predictors and the primary binary outcome, reporting adjusted ORs with 95% CIs. All analyses were performed in IBM SPSS Statistics.

The full protocol for this systematic review was registered with PROSPERO (Registration Number: CRD42022379336; https://www.crd.york.ac.uk/prospero/display_record.php?ID=CRD42022379336) and independently published^[^15^]^. This manuscript did not utilize artificial intelligence and is compliant with the TITAN guidelines^[^16^]^.

## Results

A total of 9186 unique publications were identified of which 125 met the inclusion criteria. Data extraction revealed 151 patients with a confirmed diagnosis of GA, the majority of whom were female (66%). The median age of the patients was 45 years. Supplemental Digital Content Table 1, available at: http://links.lww.com/MS9/B189 shows the characteristics of all cases and publications included in this review.

Patients with GA presented with the following signs and symptoms: right upper quadrant pain (66%), nausea (32%), emesis (30%), and jaundice (23%). Some patients had elevated liver function test results on presentation, including alkaline phosphatase (ALP, 23%), total bilirubin (20%), alanine aminotransferase (ALT, 15%), aspartate aminotransferase (AST, 15%), and gamma-glutamyl transferase (GGT, 13%). Ultrasonography was the most frequently used imaging modality (81%). Most of the patients (79%) underwent invasive operative interventions. Of those who underwent the intervention, 49.6 (59/119) underwent laparoscopy, while 50.4 (60/119) underwent an open procedure. Almost three-quarters (73%) of the GA cases were diagnosed intraoperatively. The postoperative complication rate was 8.6% (Table 2). The range of associated fetal anomalies was broad and included conditions such as hypoplastic left heart syndrome, tetralogy of Fallot, patent foramen ovale, polycystic kidney disease, biliary atresia, esophageal and rectovaginal fistulas, choledochal cysts, imperforate anus, renal agenesis, ureteral duplication, a common atrium, absent left lung, atrial septal defects as well as cleft lip and palate.

**Table 2:** Clinical features of patients with gallbladder agenesis.

Characteristics of patients with gallbladder agenesis	*n* (%)
Median age at diagnosis	45 years
Gender	
Female	98 (65)
Male	50 (33)
Unknown	3 (2)
Patient’s country of origin	
Non-US study	111 (74)
US study	40 (26)
Gallbladder agenesis diagnosed after birth	145 (96)
Other congenital anomalies	25 (17)
Incidental finding	10 (6.6)
Common symptoms	
Right upper quadrant pain	99 (66)
Nausea	49 (32)
Emesis	45 (30)
Jaundice	35 (23)
Fever	22 (15)
Dyspepsia	21 (14)
Most common laboratory findings	
Elevated ALP	34 (23)
Elevated Bilirubin	30 (20)
Elevated ALT	23 (15)
Elevated AST	22 (15)
Elevated GGT	19 (13)
Elevated WBC	18 (12)
Diagnostic imaging modality	
Ultrasound	123 (81)
Magnetic resonance cholangiopancreatography	62 (41)
Computed tomography scan	60 (40)
Endoscopic retrograde cholangiopancreatography	31 (21)
Cholangiogram	25 (17)
Hepatobiliary iminodiacetic acid scan	14 (9.3)
Magnetic resonance imaging	13 (8.6)
Surgical interventions performed	
No intervention	32 (21)
Laparoscopic surgery	59 (49.6)
Open surgery	60 (50.4)
Timing of GA diagnosis	
Intraoperatively	109 (73)
Preoperatively	41 (27)
Unknown	1
Complications	13 (8.6)

US, United States; ALP, alkaline phosphatase; ALT, alanine aminotransferase; AST, aspartate aminotransferase; GGT, gamma-glutamyl transferase; WBC, white blood cell.

There were more complications in the reported cases from the United States than in the non-US cohort, with five perioperative deaths (20% vs. 4.5%, *P* = 0.006). A preoperative diagnosis of GA was more likely to occur in recent case-studies (2013–2022), in patients with elevated liver enzyme levels, and in patients who underwent cross-sectional imaging using CT or magnetic resonance imaging (MRI) (Table 3).

**Table 3:** Comparison of clinical presentation, laboratory abnormalities, diagnostic workup, and surgical management among reported cases of gallbladder agenesis.

Comparison by intervention status, *n* (%)	Yes, *n* = 32	No, *n* = 119	*P*-value
Elevated ALP	13 (41)	21 (18)	0.006
Elevated bilirubin	12 (38)	18 (15)	0.005
Elevated GGT	8 (25)	11 (9.2)	0.031
Computer tomography scan	20 (63)	40 (34)	0.003
Magnetic resonance cholangiopancreatography	25 (78)	37 (31)	<0.001
Hepatobiliary iminodiacetic acid scan	7 (22)	7 (5.9)	0.012
Magnetic resonance imaging	6 (19)	7 (5.9)	0.032
Laparoscopic surgery	0 (0)	59 (50)	<0.001
Open surgery	0 (0)	60 (50)	<0.001
**Comparison by morbidity**	**Yes, *n* = 13**	**No, *n* = 138**	***P*-value**
Patient country of origin			0.006
Non-US study	5 (38)	106 (77)	
US study	8 (62)	32 (23)	
Ultrasound	6 (46)	117 (85)	0.003
Alive			<0.001
Yes	0 (0)	138 (100)	
Unknown	8	0	
**Comparison by timing of diagnosis**	**Preoperative *n* = 41**	**Intraoperative *n* = 109**	***P*-value**
Year of publication			0.005
Before 2000	4 (9.8)	36 (33)	
2001–2012	12 (29)	34 (31)	
2013–2022	25 (61)	39 (36)	
Age (Median [Q1–Q3] in years)	35 (21, 56)	45 (33, 62)	0.026
Patient country of origin			0.018
Non-US study	36 (88)	75 (69)	
US study	5 (12)	34 (31)	
Symptom			
Nausea	8 (20)	41 (38)	0.035
Paraclinical findings			
Elevated ALP	14 (34)	20 (18)	0.039
Elevated bilirubin	14 (34)	16 (15)	0.008
Elevated GGT	9 (22)	10 (9.2)	0.036
Computer tomography scan	25 (61)	35 (32)	0.001
Magnetic resonance cholangiopancreatography	30 (73)	32 (29)	
Type of intervention			
Open surgery	7 (17)	52 (48)	<0.001
Laparoscopic surgery	2 (4.9)	57 (52)	<0.001
No intervention	32 (78)	0 (0)	<0.001

ALP, alkaline phosphatase; ALT, alanine aminotransferase; AST, aspartate aminotransferase; GGT, gamma-glutamyl transferase.

Table summarizes clinical, laboratory, and diagnostic characteristics observed in 151 published cases of gallbladder agenesis, comparing patients who underwent intervention (laparoscopic or open surgery) to those managed without intervention, and separately comparing cases by morbidity and timing of diagnosis (preoperative vs intraoperative). Data are presented as *n* (%) unless otherwise indicated. *P*‑values were calculated using Fisher’s exact test.

Preoperative diagnosis significantly reduced surgical intervention and complications (OR 0.06, *P* = 0.044). Despite a preoperative diagnosis, 22% of patients (9/41) underwent operative interventions. Ultrasonographic diagnosis was associated with fewer complications (OR 0.10, *P* = 0.003). Higher complication rates (OR 8.53, *P* = 0.026) were associated with the use of hepatobiliary iminodiacetic acid (HIDA) scans although this likely reflects confounding by indication, since patients with more severe or diagnostically challenging presentations are often selected for HIDA imaging (Table 4).

**Table 4:** Logistic regression modeling with morbidity complication of gallbladder agenesis.

Characteristic	OR	95% CI	*P*-value
Time of diagnosis			
Intraoperative	1	—	—
Preoperative	0.06	0.00–0.56	0.044
Ultrasound			
No	1	—	—
Yes	0.10	0.02–0.42	0.003
Hepatobiliary iminodiacetic acid scan			
No	1	—	—
Yes	8.53	1.21–59.1	0.026
Elevated ALP			
No	1	—	—
Yes	3.24	0.59–17.3	0.2
Elevated ALT			
No	1	—	—
Yes	2.30	0.32–15.9	0.4

OR, odds ratio; CI, confidence interval; ALP, alkaline phosphatase; ALT, alanine aminotransferase.

## Discussion

This systematic review of 151 clinical cases of GA provides the most comprehensive analysis on presentation, management, and outcomes to date. Our findings indicate that the condition predominantly affects women, with a median age of 45 years. Most patients present with symptoms suggestive of biliary pathology. Despite the availability of various imaging modalities, the diagnosis is most commonly made intraoperatively. Notably, patients with elevated liver enzyme levels and those who underwent cross-sectional imaging (CT or MRI) were less likely to require operative intervention. However, at least 22% of the patients still underwent surgical procedures despite preoperative imaging suggesting an absence of the gallbladder. This highlights the importance of meticulous review of preoperative imaging by surgeons to avoid unnecessary operative intervention.

### Clinical presentation

Patients with GA commonly present with right upper quadrant pain, nausea, vomiting, and symptoms consistent with a biliary pathology. Although jaundice, fever, and dyspepsia were also observed, these findings are not universally present. Laboratory markers, such as ALP, AST, ALT, GGT, bilirubin, and white blood cell count, were elevated in a subset of patients. Notably, only 23% of the patients had elevated ALP levels. Given its variable presentation, definitive diagnosis relies on imaging, which has improved over time with advancements in imaging technology.

### Practical implications

Cross-sectional imaging plays a crucial role in reducing misdiagnosis of GA. Surgeons must personally review preoperative imaging, particularly ultrasonography, to confirm the presence or absence of the gallbladder before proceeding with an operation. Our findings emphasize that despite a confirmed preoperative diagnosis, at least 22% of the patients still underwent operative intervention. This suggests a failure to adequately review the imaging studies. In only one case, surgery was performed for an unrelated surgical issue, a choledochal cyst^[^17^]^.

### Imaging and misdiagnosis

Misdiagnosis of GA by imaging has been commonly reported. Ultrasound often misinterprets the absence of a gallbladder as “a gallbladder filled with hyperechoic gallstones with posterior acoustic shadowing”^[^18^]^ or described “difficult visualization of the gallbladder”^[^19^]^. MRI misinterpretations included diagnoses of “acute cholecystitis,” “sclerotic gallbladder,” or “atrophic gallbladder”^[^19^]^. These findings demonstrate that misdiagnosis is possible across all the imaging modalities.

Our review also found that the use of HIDA scans, which can yield false-positive results suggesting cystic duct occlusion, was associated with a higher complication rate. Since a positive HIDA scan cannot distinguish between an obstructed cystic duct and a completely absent gallbladder, reliance on ultrasonography or cross-sectional imaging is essential for accurate diagnosis. Patients who did not undergo surgery had significantly higher ALP, bilirubin, and GGT levels, suggesting that these markers may prompt further imaging and facilitate an early diagnosis. Further research is needed to confirm this relationship.

### Intraoperative diagnosis and management

Since GA is often diagnosed intraoperatively as our results indicate, surgeons must avoid unnecessary exploration of the biliary system in search of a non-existent gallbladder. This contrasts with the recommendation of Bani-Hani, who advocated a thorough intraoperative search for an ectopic gallbladder^[^13^]^. Although ectopic gallbladders may be located intrahepatically, retrohepatically, retroduodenally, retropancreatically, or within the retroperitoneum, we believe that an extensive intraoperative search for a “missing” gallbladder substantially increases the risk of injury to the duodenum, common bile duct, and portal vein and should therefore be strongly discouraged.

In contrast, intraoperative ultrasonography has been well-documented as an effective tool for reducing complications during cholecystectomy. Röthlin *et al* showed that laparoscopic ultrasonography was as accurate as intraoperative cholangiography for the detection of bile duct stones and variations in biliary anatomy^[^20^]^. We strongly recommend the use of intraoperative ultrasonography in intraoperative cases where the diagnosis of GA remains uncertain. In cases suspected preoperatively, cross-sectional imaging should be used to confirm the diagnosis, thereby preventing unnecessary surgical intervention.

### Geographic variation in outcomes

Interestingly, our review found that the cases reported in the United States had a higher complication rate. The reasons for this are unclear but may be related to regional variations in the management of biliary disease. Csikesz *et al* demonstrated an increase in laparoscopic cholecystectomy rates for acute cholecystitis from 83% in 1998 to 93% in 2005, reflecting an aggressive surgical approach in the United States^[^21^]^. This predisposition toward surgical intervention may contribute to the higher morbidity observed in patients with GA. Further investigation is necessary to assess the impact of regional practice patterns on patient outcomes.

### Study limitations

Our study has several limitations. The review included only articles published in English, French, or Spanish, potentially introducing a selection bias. The total number of published cases was small and together with missing data, it limited our ability to conduct a more comprehensive statistical analysis. Moreover, we did not assess the impact of evolving imaging technologies on management and outcomes. Future studies should explore the role of advanced imaging techniques in improving the preoperative diagnosis and reducing intraoperative surprises. Our findings may be influenced by publication bias, as more severe or clinically complex cases of GA are more likely to be reported, while milder or asymptomatic cases may go unrecognized or unpublished. Consequently, the literature may overrepresent diagnostic difficulties and surgical interventions, limiting the generalizability of our results. Adjusted analyses in this study are inherently constrained by the sparse and heterogeneous nature of case-level data derived from published reports and should therefore be interpreted as hypothesis-generating rather than confirmatory.

In addition, the certainty of evidence in this review is considered very low, as it is derived exclusively from case reports and small case series, which are inherently limited by reporting variability, selection bias, and incomplete clinical information. Although we employed a confirmation-based inclusion strategy to ensure diagnostic accuracy, this approach does not replace a formal, standardized risk-of-bias assessment, and the absence of such an appraisal framework represents a methodological limitation of this study. However, given the extreme rarity of GA and the lack of higher-level study designs, this approach was necessary to ensure that all available, clinically confirmed cases were captured. Our intent was not to estimate causal effects but rather to provide the most comprehensive retrospective characterization of how affected patients have presented, how they were evaluated, and whether they underwent operative intervention. This strategy allowed us to identify meaningful patterns in clinical and diagnostic features despite the recognized limitations of the underlying evidence. Our study includes all cases published between 1947 and November 2022; cases that may have been published after this period were not captured and may limit the completeness of the available evidence. Despite these findings, our study provides the most comprehensive analysis yet of the presentation, management, and outcomes in patients with GA.

## Conclusions

In summary, GA often presents with symptoms consistent with biliary pathology but is frequently misdiagnosed preoperatively, leading to unnecessary operative interventions and increased complication rates. A proper review of the preoperative imaging by the operating surgeon is essential to prevent unnecessary surgical interventions. Cross-sectional imaging remains a critical modality for accurate diagnosis of this condition, ultimately improving patient outcomes and reducing healthcare costs.

## Data Availability

Studies included have been shared as Supplemental Digital Content Table 1, available at: http://links.lww.com/MS9/B189. All other data available upon reasonable request.

## Research registration unique identifying number (UIN)

*CRD42022379336;* https://www.crd.york.ac.uk/prospero/display_record.php?ID=CRD42022379336)
